# Subclinical left ventricular systolic impairment in hypertensive patients: insights from 2d speckle-tracking echocardiography

**DOI:** 10.1038/s41371-025-01100-x

**Published:** 2025-12-15

**Authors:** Mohammad Shafiq, Mostafa M. Hassan, Yasser AbdelHady

**Affiliations:** 1 Cardiology department, Faculty of medicine, Beni-Suef University, Beni-Suef, Egypt; 2 Cardiology department, Insurance Hospital, Beni-Suef, Egypt

**Keywords:** Hypertension, Echocardiography

## Abstract

Cardiovascular diseases, the leading global cause of mortality, are frequently driven by hypertension, which contributes to left ventricular hypertrophy (LVH) and heart failure. Although ejection fraction (EF) remains the standard metric for assessing left ventricular function, it often fails to detect early dysfunction. Speckle-tracking echocardiography (STE) provides a more sensitive approach to identifying subclinical myocardial strain changes before EF declines. This cross-sectional, case-control study evaluated 90 participants divided into three groups: 30 healthy controls (Group I), 40 hypertensive patients without LVH (Group II), and 20 hypertensive patients with LVH (Group III). All underwent clinical evaluation, conventional echocardiography, and 2D-STE. Results showed that Group III had significantly greater septal and posterior wall diastolic thickness (p = 0.001) and higher LV mass index (p = 0.001) compared to Groups I and II. 2D-STE revealed reduced apical (AP2Ls, AP3Ls) and global longitudinal strain (GLS) in Groups II and III versus controls. Basal and mid-segment strains were also lower in disease groups, with mid-inferolateral segments showing more pronounced impairment in Group III. These findings highlight that Group III exhibited the most severe structural and functional cardiac alterations. The study demonstrates the superiority of 2D-STE over conventional echocardiography in detecting subclinical LV dysfunction in hypertensive patients, particularly those with LVH, through impaired longitudinal strain measurements. Early integration of 2D-STE into clinical practice could facilitate timely interventions to mitigate myocardial remodeling and heart failure progression.

## Introduction

Cardiovascular diseases are the leading cause of global mortality. Arterial hypertension is a major risk factor for cardiovascular morbidity and mortality worldwide [1]. Chronic hypertension induces left ventricular hypertrophy (LVH), an early sign of organ damage caused by high blood pressure [2]. LVH directly predisposes to and exacerbates irreversible deterioration of LV function, ultimately progressing to congestive heart failure [3, 4]. Consequently, detecting LVH is important for assessing one’s overall cardiovascular risk [2].

The current gold standard echocardiographic parameter of LV systolic function remains ejection fraction (EF) [5]. However, EF measurement represents an oversimplified approach that primarily reflects radial myocardial deformation while neglecting the complex three-dimensional deformation patterns, particularly in the longitudinal and circumferential planes. Consequently, EF assessment only identifies LV functional impairment at relatively advanced stages [6].

Subclinical alterations in myocardial deformation precede the more pronounced LV dysfunction detectable by EF changes. Experimental studies utilizing isolated papillary muscle preparations under chronic pressure overload conditions have demonstrated reduced contractility despite preserved EF [7–9]. Thus, conventional methods frequently fail to detect early stages of LV functional impairment [10].

Advanced quantitative echocardiographic deformation imaging techniques, particularly speckle tracking echocardiography (STE), enable the measurement of left ventricular strain, thereby enhancing the diagnosis of hypertensive heart disease [11, 12]. STE employs specific physical principles for strain quantification, utilizing two-dimensional grayscale B-mode imaging [13]. In various clinical conditions where conventional indices often remain within normal limits, 2D-STE has proven useful in detecting discrete reductions in myocardial contractility and regional mechanics, thereby serving as a sensitive indicator of latent LV dysfunction [14]. Compared to ejection fraction assessment, STE demonstrates superior sensitivity in detecting subclinical alterations in left ventricular deformation [15, 16].

In our study, we tested the utility of STE in detecting subtle cardiac dysfunction in patients with hypertension. With the use of STE, we elaborated on the value of measures of myocardial systolic strain in detecting subtle LV systolic deformation abnormalities in hypertensive patients with preserved EF at rest.

## Methods

In accordance with the STROBE checklist, we reported the following cross-sectional case-control study. Conducted at Beni-Suef University Hospital (March 2019-February 2023), 90 patients were divided into three groups. Group I (controls) included 30 age- and sex-matched individuals without apparent cardiovascular risk factors. Group II included 40 patients with hypertension and no evidence of LVH (HTN-LVH group). Group III included 20 patients with hypertension and LVH (HTN+LVH group).

We included hypertensive patients, i.e., those with a systolic blood pressure of or more than 140 mmHg ± diastolic blood pressure of 90 mmHg or more on at least two separate occasions of attended office measurements or those who were previously diagnosed with hypertension and were maintained on antihypertensive medications, although drug data was not collected for our analysis. We excluded those who had diabetes, had hypertension-mediated organ affection other than LVH, had compromised LV function (EF < 50% or symptomatic heart failure), stroke, or were known ischemic heart disease patients (previous angina, MI, PCI, CABG, etc.). In addition, we excluded patients with arrhythmias, severe valve lesions, chronic obstructive pulmonary disease (COPD), as well as those with pericardial or congenital heart disease.

Our patients underwent comprehensive history taking, physical evaluation, and investigations during their first visit. Under optimal conditions, blood pressure was measured using a sphygmomanometer. A resting electrocardiogram (ECG) was done for all participants to identify those with ischemic changes, rhythm disturbance, and exclude them. In addition, the Cornell criteria were used to diagnose LVH [17], and the diagnosis was then verified by the modified Cornell Criteria [18] and the Sokolow-Lyon Criteria [19]. Lastly, a score of 4–5 on the Romhilt-Estes LVH Point Score System was used to consolidate the diagnosis further [20].

Conventional transthoracic echocardiography and 2D-STE were performed using a Philips USA EPIQ 7 ultrasound machine for all participants, while they were in the left lateral decubitus position and connected to a single-lead ECG.

### Conventional echocardiography

The dimensions of the LV were assessed via the parasternal long-axis M-mode, measuring the interventricular septal diastolic thickness (IVSd), LV posterior wall diastolic thickness (PWd), LV diastolic diameter (LVDd), and LV systolic diameter (LVDs). Based on those, we estimated the LV mass [21], and the LVM index was derived from it in relation to body surface area (LVM/BSA, g/m²). Cutoff points to identify LVH were set at 115 g/m for men and 95 g/m for women [22]. This was then used to establish the diagnosis of LVH.

The modified biplane method was utilized to evaluate the LV systolic function, LVED volume, and LVES volume from both the apical 4- and 2-chamber views. EF was then calculated [23].

### Speckle tracking echocardiography (2D-STE)

#### Image acquisition

**Fig. 1:**
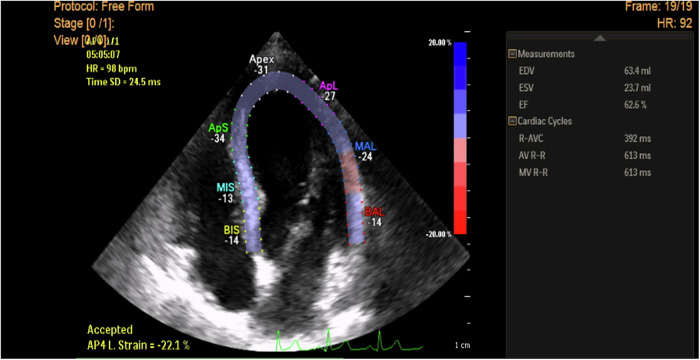
2D STE measurement of longitudinal strain in apical 4-ch view in a hypertensive patient without LVH shows a reduction of peak LS of basal segments of septal &lateral walls (Esys = −14%) but normal peak longitudinal strain of the remaining myocardial segments and normal global LV strain (GS = - 22.1%).

**Fig. 2:**
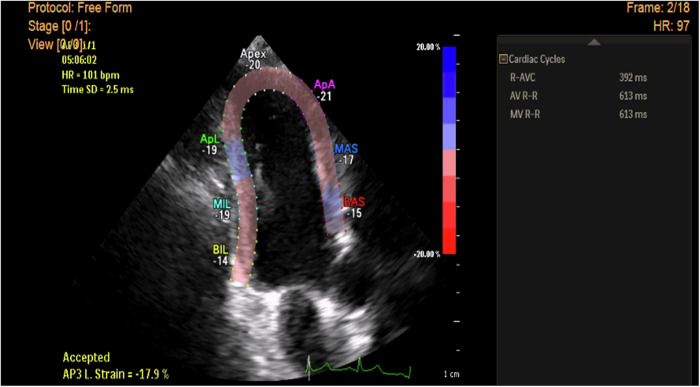
2D STE measurement of longitudinal strain in apical 3-ch view in a hypertensive patient with LVH showing reduction of peak longitudinal strain value of basal & mid segments of anteroseptal wall & basal posterior wall (Esys = −15 - 17, −14% respectively) but normal peak longitudinal strain of the remaining myocardial segments.

The apical 4-, 2-, and 3-chamber echocardiographic views were captured at a frame rate of 60–110 frames per second. Three consecutive ECG-gated cardiac cycles are recorded during end-expiratory breath-holding to ensure stable electrocardiographic (ECG) signals and minimize motion artifacts. Care is taken to avoid shortening the appearance of the ventricle and to clearly visualize the endocardial border, ensuring high-quality images. These images are stored for later offline analysis (Figs. 1–2**)**.

#### Timing of cardiac events

The timing of events, such as aortic valve closure (AVC), is determined either visually (using the apical long-axis view) or semi-automatically.

### Image analysis process

**Fig. 3:**
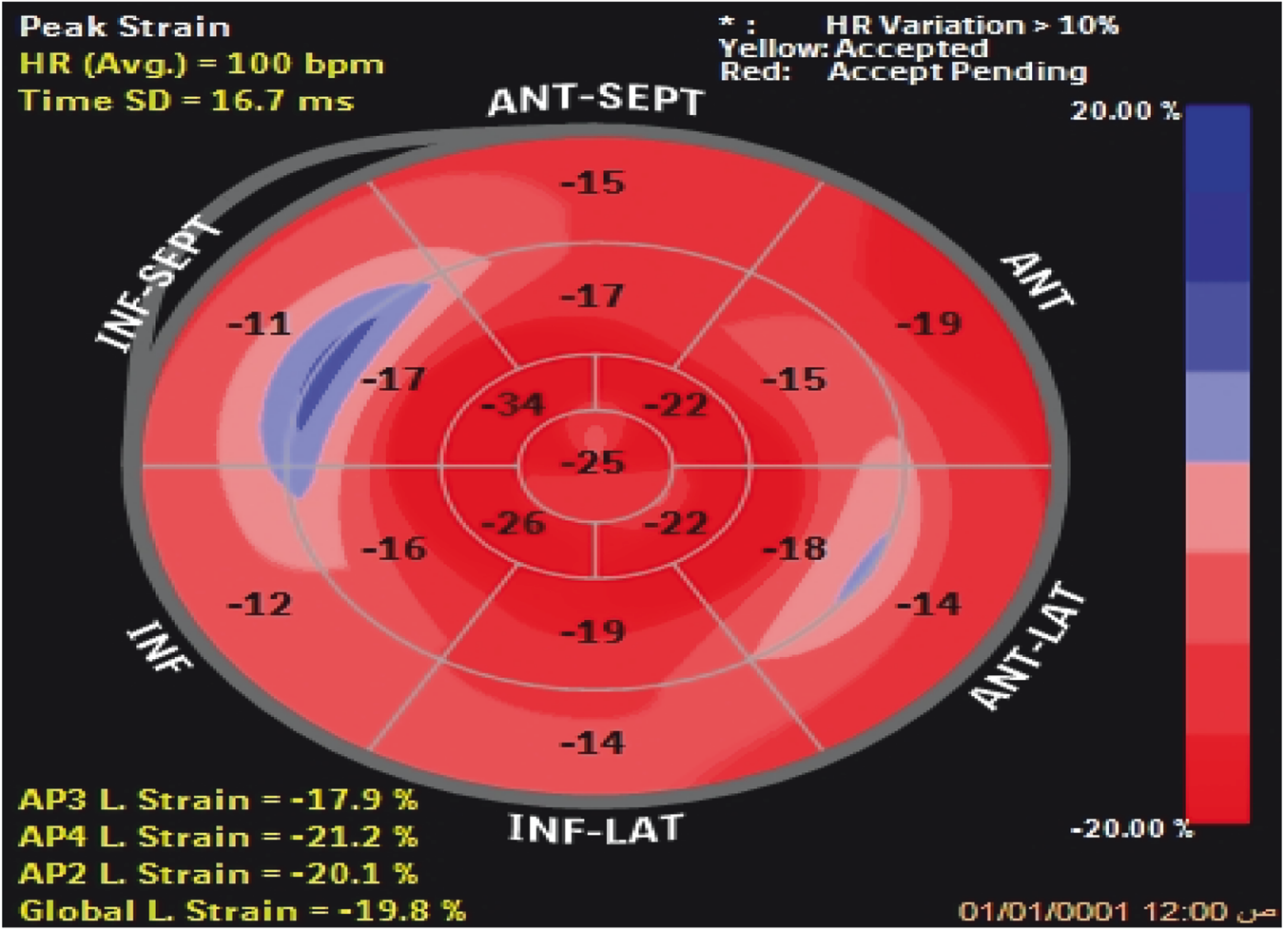
Bull’s eye projections of peak longitudinal strain of 17 myocardial segments in a hypertensive patient with LVH showing marked reduction of peak systolic strain of all basal myocardial segments except basal anterior walls & marked reduction of peak systolic strain of all mid myocardial segments except mid inferior wall.

1. **Strain Measurement**: Offline analysis is performed using semiautomatic computer software (2D Speckle Tracking Imaging, STI) to calculate strain and strain rate.
  We manually drew the region of interest (ROI) along the endocardial border.
  Automatically, the epicardial border was traced by the software, though wall thickness could be manually adjusted.
  At end-diastole, we defined the ROI using three contours: the endocardial and epicardial borders and the myocardial midline.
2. **Global and Regional Analysis:**
  GLS was calculated as the average longitudinal strain across all myocardial segments in all views.
  The myocardium is divided into a standardized 17-segment model (displayed as a bull’s-eye plot, Fig. 3) for detailed regional assessment. Longitudinal strain is evaluated in the six LV walls, and the LV is then segmented automatically into the 17 regions. Strain values from the 17 LV segments were calculated and compared between the control and patient groups to identify differences in myocardial function.

### Statistical analysis

All data were analyzed using SPSS v. 25. For categorical data, the Chi-square test was used. For normally distributed data between two groups, we used the Student t-test, whereas the ANOVA test was used to compare these data among all three groups. Normality was assessed using the Kolmogorov test. Statistical significance was deemed to be at a p-value of ≤ 0.05.

### Ethical considerations

We ensured that our study aligned with the ethical tenets of the Declaration of Helsinki. All research procedures were conducted with respect for the rights, dignity, and well-being of our participants. Informed consent was secured before enrollment, and all personal data were anonymized to ensure confidentiality. Ethical approval was obtained from the Beni-Suef University Higher Research Ethics Committee (BSU-HREC).

## Results

**Table** 1**: Demographics and baseline characteristics:**

**Table 1:** Demographics and baseline characteristics.

	Group I (control) 30 patients	Group II (HTN without LVH) 40 patients	Group III (HTN with LVH) 20 patients	Tukey’s post hoc test		
				P value Group I: Group II	P value Group I: Group III	P value Group II: Group III
Age (years) mean ± SD	58.73 ± 7.72	60.15 ± 6.55	59.80 ± 6.13	0.125	0.107	0.981^*^
Systolic blood pressure (mmHg) mean ± SD	113.83 ± 13.31	137.88 ± 12.86	142.00 ± 18.24	<0.001	<0.001	0.547^*^
Diastolic blood pressure (mmHg) mean ± SD	68.33 ± 8.02	86.25 ± 10.300	86.75 ± 12.59	<0.001	<0.001	0.982^*^
Sex (male) No. (%)	17 (56.7%)	20 (50%)	10 (50%)	0.837 **		
Hypertension duration (years) mean ± SD		10.06 ± 4.52	10.60 ± 3.89			0.205^***^

^*^ANOVA test.

**Chi-Square test.

***student t-test, level of significance ≤0.05.

Our study initially screened 106 patients but excluded 16 due to ECG findings indicative of ischemia or arrhythmia. The remaining 90 participants were divided into three groups, with a mean age of 58.5 ± 9.5 years. The three groups were similar in age and sex distribution. However, both groups with HTN showed statistically significant differences in systolic and diastolic blood pressure compared to the control group. The duration of hypertension (2–18 years) was similar across the two hypertension groups (see Table 1).

**Table** 2**: Conventional Echocardiographic Parameters:**

**Table 2:** comparison of conventional echocardiographic findings between the studied groups.

	Group I (control) 30 patients	Group II (HTN without LVH) 40 patients	Group III (HTN with LVH) 20 patients	Tukey’s post hoc test*		
				P value Group I: Group II	P value Group I: Group III	P value Group II: Group III
Septal wall diastolic thickness (SWTd) mean ± SD	0.82 ± 0.07	0.83 ± 0.07	1.29 ± 0.03	0.764	***<0.001***	***<0.001***
Left ventricular end systolic diameter (LVEDs) mean ± SD	3.06 ± 0.25	3.08 ± 0.29	3.15 ± 0.27	0.895	0.498	0.707
Left ventricular end diastolic diameter (LVEDd) mean ± SD	4.64 ± 0.33	5.64 ± 6.08	4.67 ± 0.22	0.567	0.999	0.664
Posterior wall diastolic thickness (PWTd) mean ± SD	0.79 ± 0.08	0.78 ± 0.08	1.27 ± 0.08	0.886	***<0.001***	***<0.001***
Fractional shortening (FS %) mean ± SD	33.37 ± 3.21	33.27 ± 3.54	32.85 ± 2.08	0.992	0.839	0.877
Ejection fraction (EF%) mean ± SD	62.83 ± 3.29	62.48 ± 3.89	61.94 ± 3.53	0.915	0.669	0.847
Left ventricular mass index mean ± SD	85.40 ± 7.37	86.73 ± 8.24	120.80 ± 7.78	0.765	***<0.001***	***<0.001***

^*^ANOVA test, level of significance ≤0.05.

*P* values in bold represent the highly significant difference between the column groups regarding the row values.

Septal wall diastolic thickness (SWTd) was significantly greater in the HTN + LVH group compared to both controls (0.82 ± 0.07 vs. 1.29 ± 0.03, p = 0.001) and HTN-LVH (0.83 ± 0.07 vs. 1.29 ± 0.03, p = 0.001). Similarly, posterior wall diastolic thickness (PWTd) was greater in HTN + LVH than in controls (0.79 ± 0.08 vs. 1.27 ± 0.08, p = 0.001) and the HTN-LVH group (0.78 ± 0.08 vs. 1.27 ± 0.08, p = 0.001). However, when comparing HTN-LVH to controls, we found no significant differences in SWTd or PWTd.

No significant differences were observed among the three groups in LVEDs, LVEDd, FS%, or EF%. Left ventricular mass index (LVMI) was significantly larger in the group with LVH compared to both controls (85.40 ± 7.37 vs. 120.80 ± 7.78, p = 0.001) and HTN-LVH (86.73 ± 8.24 vs. 120.80 ± 7.78, p = 0.001) (Table 2).

**Table** 3**: 2D-STE Findings:**

**Table 3:** comparison of 2D Speckle tracking echocardiographic findings between the studied groups.

	Group I (control) 30 patients	Group II (HTN without LVH) 40 patients	Group III (HTN with LVH) 20 patients	Tukey’s post hoc test*		
				P value Group I: Group II	P value Group I: Group III	P value Group II: Group III
**Apical systolic longitudinal strain (APLs)**						
**-**AP2Ls	−25.0 ± 2.98	−20.48 ± 3.12	−19.54 ± 5.65	< ***0.001***	< ***0.001***	0.633
-AP3Ls	24.96 ± 3.01	−19.99 ± 2.29	−20.99 ± 4.97	< ***0.001***	< ***0.001***	0.514
-Ap4Ls	21.16 ± 1.42	−20.80 ± 2.75	−19.37 ± 1.42	0.755	***0.011***	***0.04***
-Global Ls	24.97 ± 3.01	−20.60 ± 2.88	−20.15 ± 5.31	***<0.001***	< ***0.001***	0.889
**Basal segment systolic longitudinal strain**						
-Anterior	−18.98 ± 1.62	−16.45 ± 2.69	−14.95 ± 2.79	< ***0.001***	< ***0.001***	0.067
-Anterolateral	−17.43 ± 2.73	−15.73 ± 3.97	−13.95 ± 3.58	0.118	***0.003***	0.16
-Inferolateral	−17.36 ± 3.05	−14.43 ± 4.25	−12.400 ± 3.94	***0.006***	< ***0.001***	0.135
-Inferior	−18.82 ± 1.77	−17.20 ± 2.73	−15.55 ± 3.10	***0.028***	< ***0.001***	0.053
-Inferoseptal	−21.20 ± 1.49	−19.37 ± 2.13	−17.85 ± 2.11	***0.001***	< ***0.001***	***0.014***
-Anteroseptal	−21.16 ± 1.42	−19.71 ± 2.15	−18.18 ± 1.86	***0.005***	< ***0.001***	***0.010***
**Mid segment systolic longitudinal strain**						
**-**Anterior	−20.71 ± 1.91	−19.09 ± 1.98	−18.14 ± 1.51	***0.002***	< ***0.001***	0.153
-Anterolateral	−20.72 ± 1.89	−19.25 ± 1.92	−18.29 ± 1.43	***0.004***	***<0.001***	0.136
-Inferolateral	−20.71 ± 1.91	−18.61 ± 2.58	−17.00 ± 2.31	***0.001***	***<0.001***	***0.035***
-Inferior	−17.62 ± 2.52	−15.49 ± 3.11	−15.25 ± 3.16	***0.010***	***0.018***	0.951
-Inferoseptal	−20.71 ± 1.91	−18.95 ± 2.11	−18.29 ± 1.50	***0.001***	< ***0.001***	0.42
-Anteroseptal	−18.82 ± 1.77	−17.47 ± 2.21	−16.60 ± 2.37	***0.027***	***0.001***	0.288
**Apical segment systolic longitudinal strain**						
**-**Anterior	−21.23 ± 1.46	−19.44 ± 1.91	−18.60 ± 1.72	< ***0.001***	< ***0.001***	0.183
-Septal	−24.96 ± 3.01	−22.79 ± 4.21	−20.88 ± 5.05	0.076	***0.002***	0.205
-Inferior	−25.00 ± 2.98	−22.35 ± 3.73	−20.90 ± 5.03	***0.015***	***0.001***	0.357
-Lateral	−20.68 ± 1.94	−18.75 ± 2.39	−17.45 ± 1.93	***0.001***	< ***0.001***	0.075
-Apex	−21.20 ± 1.42	−19.73 ± 2.21	−17.94 ± 2.02	***0.007***	***<0.001***	***0.003***

*ANOVA test, level of significance ≤0.05.

*P* values in bold represent the highly significant difference between the column groups regarding the row values.

Regarding global and apical longitudinal strain measurements, AP2Ls and AP3Ls were significantly higher in the control group vs. Groups II and III (p < 0.001). No differences were noted between the hypertensive groups in terms of these measures (p = 0.633, p = 0.514). AP4Ls showed no significant differences between HTN-LVH and controls (p = 0.755), but statistically significant differences were noted between HTN + LVH and controls (p = 0.011) as well as between both hypertensive groups (p = 0.04). GLS was significantly higher in the control group as opposed to the two hypertensive groups (p < 0.001), though comparisons drawn between the two hypertensive groups were not significantly different (p = 0.889).

Evaluating the basal systolic longitudinal strains revealed that the basal LS of the anterior segment was higher in the control group vs. both hypertensive groups (p < 0.001), while both groups with hypertension ± LVH were comparable (p = 0.067). In addition, the anterolateral segment basal LS was higher in the control group vs. HTN + LVH (p = 0.003), with no differences between control vs. HTN-LVH, or HTN-LVH vs. HTN + LVH. Inferolateral, inferior, inferoseptal, and anteroseptal segment basal LS were significantly higher in the control group compared to HTN-LVH (p = 0.028, p = 0.001, p = 0.005, respectively) and HTN + LVH (p < 0.001 for all). Inferoseptal and anteroseptal basal LS were also higher in Group HTN-LVH compared to HTN + LVH (p = 0.014, p = 0.010).

We addressed the mid-systolic LS and found that the anterior, anterolateral, inferolateral, inferior, inferoseptal, and anteroseptal segments were all higher in the control group vs. HTN-LVH (p = 0.002, p = 0.004, p = 0.001, p = 0.010, p = 0.001, p = 0.027, respectively) and HTN + LVH (p < 0.001). The inferolateral segment demonstrated higher values in the group without LVH vs. HTN + LVH (p = 0.035), whereas the anterior, anterolateral, inferior, inferoseptal, and anteroseptal segments showed no differences between HTN-LVH and HTN + LVH (p > 0.05).

Lastly, the apical segment LS was as follows: Anterior, inferior, lateral, and apical segments were higher in the control group compared to both hypertensive groups (p < 0.05). Apical septal LS did not differ between control and those without LVH (p = 0.076). Apical segment LS was higher in those without LVH as opposed to those with LVH (p = 0.003). Anterior, septal, inferior, and lateral segments did not significantly vary between both hypertensive groups (p > 0.05) (Table 3).

**Table** 4**: Correlation between LVMI and longitudinal strain parameters in each group:**

**Table 4:** Correlation between left ventricular mass index and longitudinal strain parameters in each group.

Groups		AP2Ls	AP3Ls	AP4Ls	GLs
**Group I (LVMI)**	**r**^**2**^	0.016	−0.007	−0.197	−0.007
	**P value**	0.935	0.969	0.296	0.969
**Group II (LVMI)**	**r**^**2**^	0.169	0.272	0.135	0.163
	**P value**	0.298	0.089	0.406	0.316
**Group II (LVMI)**	**r**^**2**^	−0.344	−0.302	−0.111	−0.332
	**P value**	0.138	0.196	0.641	0.153

*LVMI* left ventricular mass index, *AP2L* apical longitudinal strain, *Gls* global longitudinal strain.

In the control group, no significant correlation was observed between LVMI and AP2Ls, AP3Ls, AP4Ls, or Global Ls (p > 0.05). Hypertensive patients with or without LVH did not demonstrate any meaningful correlations between LVMI and all apical strains (AP2Ls, AP3Ls, AP4Ls, Global Ls; p > 0.05; Table 4).

**Table** 5**: Correlation between different conventional echocardiographic parameters and GLS:**

**Table 5:** Pearson correlation between different conventional echo parameters and global longitudinal strains.

		LVMI	SWTd	LVEDs	LVEDd	PWTd	FS%	EF%
**Group I (GLS)**	**r**^**2**^	−0.007	−0.028	0.28	0.351	0.243	0.202	0.156
	**P value**	0.969	0.883	0.134	0.057	0.196	0.285	0.412
**Group II (GLS)**	**r**^**2**^	0.163	0.085	0.093	***0.506***	0.134	−0.009	0.116
	**P value**	0.316	0.601	0.566	***0.001***	0.41	0.957	0.476
**Group II (GLS)**	**r**^**2**^	−0.332	0.038	0.082	0.166	0.238	0.249	0.111
	**P value**	0.153	0.875	0.732	0.486	0.313	0.289	0.642

*LVMI* left ventricular mass index, *SWTd* septal wall diastolic thickness, *LVEDs* left ventricular end systolic diameter, *LVEDd* left ventricular end diastolic diameter, *PWTd* posterior wall diastolic thickness, *FS* fractional shortening, *EF* ejection fraction.

Bold values are to highlight the significant statistical values in the table for easier recognition.

We found no plausible correlations between conventional echocardiographic parameters (LVMI, SWTd, LVEDs, LVEDd, PWTd, FS, EF%) and GLS in the control group. In the group without LVH, there was a statistically significant, moderate, positive correlation between GLS and LVEDd (r^2^ = 0.506, p = 0.001). In the group with HTN + LVH, there was no significant correlation between GLS and any of the conventional parameters (Table 5).

## Discussion

In our study, we intended to assess LV systolic function using 2D-STE in 90 patients with essential hypertension, categorized into three groups: controls (Group I), hypertensive patients without LVH (Group II), and hypertensive patients with LVH (Group III). We primarily assessed global and segmental LV systolic strain.

Regarding the septal wall thickness (SWTd), we found that those with LVH had substantially greater SWTd compared to those without LVH as well as controls (1.29 vs. 0.83 vs. 0.82, p = 0.001). No significant variation was discernible among our subjects regarding the EF or LV diameters. However, LVMI was considerably greater in those with LVH compared to the controls and hypertensive patients with no LVH (p = 0.001). In line with these findings, Sengupta et al. noted that hypertensive patients had an exceedingly greater SWT (1.5 vs. 0.8 cm, p < 0.001). Nevertheless, they found no significant differences regarding LVMI (62 vs. 53, p > 0.05) or EF (67 vs. 65, p > 0.05). The discrepancy in LVMI between our findings and theirs could be owed to their relatively smaller sample and the younger age of their cohort [24].

Results revealed that hypertensive patients with normal ejection fraction and no heart failure symptoms exhibited impaired myocardial deformation (primarily longitudinal strain) via STE, undetectable by conventional echocardiography. These findings highlight the potential of using STE for early detection of subclinical LV systolic dysfunction and mechanical alterations, surpassing the sensitivity of conventional echocardiography in identifying initial contractility changes. We confirmed via 2D-STE that hypertensive patients, both with and without LVH, exhibit early myocardial deformation changes—evidenced by reduced global and segmental longitudinal systolic strain—compared to controls. Notably, hypertensive patients with LVH (Group III) demonstrated a significant decline in longitudinal systolic strain compared to those without LVH (Group II).

Narayanan A et al. compared 52 hypertensive patients and 52 controls using 2D echocardiography, which demonstrated normal LV contractility, EF, and FS. On 2D-STE, no significant differences in circumferential, radial, or longitudinal systolic strain between groups were found. However, the study did not clarify the duration or severity of hypertension among its sample, which limited the comparability of its findings to ours. The authors suggested that velocity abnormalities, emerging early in hypertension before global strain changes, represent a critical target for preventive strategies to address subclinical dysfunction [25].

Sengupta et al. found that patients with systemic hypertension had significantly lower longitudinal strain of both the subendocardium (−13.4 vs. −20.2%, p < 0.001) and the subpericardium (−11.3 vs. −17.5%, p < 0.001). However, they did not subcategorize their hypertensive group based on the presence or absence of LVH, which limited the comparability to our findings [24]. Suggestive of these findings, Poulsen et al. noted that in hypertensive patients who were previously considered to have isolated diastolic dysfunction, tissue tracking and systolic strain measures revealed LV systolic longitudinal impairment, indicating that such subtle changes can be detected by incorporating longitudinal strain measures in risk assessments [26]. We explained this by emphasizing that the longitudinal myocardial fibers are especially prone to ischemic injury, leading to a decrease in longitudinal strain measures [27]. Jones et al. demonstrated that there is a coordinated long-axis and short-axis ventricular motion with characteristic coupling in healthy individuals, the loss of which serves as an early marker of systolic ventricular dysfunction. These motion disturbances precede alterations in conventional systolic metrics, such as FS or peak circumferential fiber shortening velocity [27].

Bello et al. corroborated our findings, as they demonstrated that there was a significant reduction in the LV longitudinal systolic strain of hypertensive and prehypertensive patients compared to the control group [28]. Consistently, Kouzu et al. found that the LV longitudinal systolic strain of hypertensive patients with LVH was substantially reduced compared to controls, with increased radial systolic strain [29]. Another study by Imbalzano et al., conducted on 51 hypertensive patients with or without LVH, and a control group of 51 individuals, found that there was impairment of global & segmental longitudinal systolic strain on 2D-STE in hypertensive patients, both those with LVH and those without [30].

Kang et al. relayed that the increased LV overload and increased end-systolic wall stress cause increased subendocardial deposition of collagen and myocardial fibrosis, which leads to a reduction of longitudinal systolic strain. This was evident in hypertensive patients with normal LV EF and decreased LV longitudinal systolic strain who demonstrated increased levels of tissue inhibitor of matrix metalloproteinase-1 (TIMP1), which is an indicator of myocardial fibrosis [31].

We aimed to explain the reduction of myocardial deformation in hypertensive patients with normal LVEF by highlighting the compensatory increase of the LV circumferential strain, with subsequent reductions in radial and longitudinal strain. This was verified by Imbalzano et al. [30]. Edvardsen et al. emphasized the limited sensitivity of conventional echocardiography in assessment of LV systolic function compared to 2D-STE measurements, as suggested by the finding of normal LV systolic function in patients with heart failure with preserved ejection fraction (HFpEF) who had presented with symptoms and signs of heart failure, yet had normal EF on routine echocardiography [32]. A study by Sjoli et al. compared myocardial function in patients with acute myocardial infarction using 2D-STE and conventional echocardiography. They found that systolic strain measurements via STE were more sensitive than left ventricular ejection fraction (LVEF) from conventional methods in detecting early LV dysfunction. This is because LVEF, a volumetric measure, relies on geometric assumptions that limit its accuracy, whereas systolic strain directly assesses myocardial deformation without dependence on LV geometry. The study also revealed that LV hypertrophy, often caused by prolonged or elevated LV afterload (e.g., hypertension), leads to structural and functional changes such as fibrosis, myocardial ischemia, altered LV wall motion, and impaired diastolic function. Specifically, the subendocardium is vulnerable to damage from interstitial fibrosis and reduced blood flow, which disrupts longitudinal contraction—a key component of systolic strain. These findings emphasize the utility of myocardial strain patterns, which reflect LV adaptation to chronic pressure overload, making strain analysis a superior tool for evaluating subtle systolic dysfunction compared to LVEF [33].

In our study, hypertensive patients with LVH displayed a significant reduction in global and segmental longitudinal strain compared to those without LVH and controls. Similarly, Saghir et al. studied 60 hypertensive patients and 48 control and found that hypertensive patients with LVH had significant reduction in global and segmental longitudinal systolic strain values, namely peak systolic strain rate (SR_S_) and peak early diastolic strain rate (SR_E_; −16.8%, −0.99 s^−1^; 1.54 s^−1^, respectively) compared with controls ( − 21.7%, −1.31 s^−1^, and 2.35 s^−1^, respectively; all p < 0.0001) [34]. These results aligned with the work of Mizuguchi et al., who found that longitudinal, radial, and circumferential systolic strains were significantly lower in the hypertensive group with concentric LVH compared with the control group. Moreover, strain measures were all significantly lower in patients with LVH compared to hypertensive patients without LVH or with eccentric LVH. This study also stated that there was a positive correlation between the progression of LVH and the progression of cardiac symptoms towards NYHA class IV, whereas worsening LVH was marked by further reduction in global longitudinal LV systolic strain [35].

Our study went beyond the broadly recognized utility of speckle tracking in merely identifying subclinical myocardial dysfunction. We used speckle tracking to compare subclinical myocardial dysfunction between two groups of hypertensive patients: those with left ventricular hypertrophy (LVH) and those without. We specifically analyzed the differences in their cardiac strain patterns, which is clinically significant because these patients are at high risk of developing heart failure with preserved ejection fraction (HFpEF) that can be diagnosed before manifest clinical HF. Nevertheless, we acknowledge several limitations that stemmed from variability in hypertension severity, duration, or treatment, as well as our small sample size and the single-center, cross-sectional design, which does not account for the long-term impacts of hypertension. Additionally, the mean SBP and DBP were estimated based on office measurements only, without considering ambulatory blood pressure measurements, which are subject to fallacious readings due to various reasons, such as the white coat effect. In addition, our participants were on antihypertensive treatment, for which complete data were not collected, which may have confounded the analysis. Some technical issues were encountered with STE, including the quality of the images and the technical difficulty in tracing the myocardial borders in tachycardic patients, as well as inherent inter-operator variability.

## Conclusion

This study detailed the pivotal role of 2D-STE in identifying subclinical LV systolic dysfunction in hypertensive patients, even when conventional echocardiography measures such as EF remain normal. By evaluating global and segmental longitudinal strain, 2D-STE revealed significant impairment in myocardial deformation—particularly in longitudinal fibers—among hypertensive patients, with the most pronounced reductions observed in those with LVH. These findings align with prior research demonstrating that longitudinal strain deteriorates early in hypertension, driven by subendocardial fibrosis, increased wall stress, and compensatory adaptations in radial and circumferential strain that mask dysfunction in conventional EF assessments. Our study emphasized the superiority of 2D-STE over traditional echocardiography in detecting subtle mechanical changes, offering a window for early intervention to mitigate progression toward overt heart failure. Thus, we advocate for integrating 2D STE into the routine evaluation of hypertensive patients, particularly those with LVH, to enable timely management of myocardial remodeling. Future studies with larger, homogenous cohorts and advanced imaging techniques are warranted to refine risk stratification and therapeutic strategies.

## Summary

### What is known about the topic

- Left ventricular hypertrophy (LVH) in systemic hypertension is a recognized precursor to asymptomatic LV dysfunction and congestive heart failure.
- The loss of ventricular longitudinal myocardial fiber motion coordination is reflected on longitudinal strain measures.
- Left ventricular diastolic dysfunction associated with LVH can be detected on conventional echocardiography, but LV systolic function is usually intact in the early stages of hypertension.

### What this study adds

- Unlike prior studies, this work categorizes patients into hypertensive without LVH and hypertensive with LVH, alongside controls. This stratification reveals a gradient of impairment, with patients with LVH having significantly worse strain reductions in specific segments.
- Our study provides data on regional myocardial deformation, identifying basal and mid-segment strains as particularly impaired in hypertensive patients, apical segments (AP2Ls, AP3Ls) and global longitudinal strain (GLS) as key discriminators between hypertensive patients and controls, and specific segments (e.g., mid inferolateral) with pronounced deterioration in LVH patients, offering targets for focused clinical monitoring.
- Functional changes may precede structural changes or occur independently of structural hypertrophy, advocating for STE as a complementary tool even in structurally intact hearts.
- By identifying subclinical strain abnormalities in hypertensive patients, particularly those with LVH, the study emphasizes the role of STE in risk stratification.

## Data Availability

The datasets generated during and/or analyzed during the current study are available from the corresponding author upon reasonable request.
